# Factors Influencing the Eicosanoids Synthesis *In Vivo*

**DOI:** 10.1155/2015/690692

**Published:** 2015-03-11

**Authors:** Jarosław Szefel, Wiesław Janusz Kruszewski, Ewa Sobczak

**Affiliations:** ^1^Department of Propaedeutic Oncology, Faculty of Health Sciences, Medical University of Gdańsk, Powstania Styczniowego 9b, 81-519 Gdynia, Poland; ^2^Department of Surgical Oncology, Gdynia Oncology Center, PCK's Maritime Hospital in Gdynia, Powstania Styczniowego 1, 81-519 Gdynia, Poland

## Abstract

External factors activate a sequence of reactions involving the reception, transduction, and transmission of signals to effector cells. There are two main phases of the body's reaction to harmful factors: the first aims to neutralize the harmful factor, while in the second the inflammatory process is reduced in size and resolved. Secondary messengers such as eicosanoids are active in both phases. The discovery of lipoxins and epi-lipoxins demonstrated that not all arachidonic acid (AA) derivatives have proinflammatory activity. It was also revealed that metabolites of eicosapentaenoic acid (EPA) and docosahexaenoic acid (DHA) such as resolvins, protectins, and maresins also take part in the resolution of inflammation. Knowledge of the above properties has stimulated several clinical trials on the influence of EPA and DHA supplementation on various diseases. However, the equivocal results of those trials prevent the formulation of guidelines on EPA and DHA supplementation. Prescription drugs are among the substances with the strongest influence on the profile and quantity of the synthesized eicosanoids. The lack of knowledge about their influence on the conversion of EPA and DHA into eicosanoids may lead to erroneous conclusions from clinical trials.

## 1. Stages of Eicosanoid Synthesis

**Figure 1:**
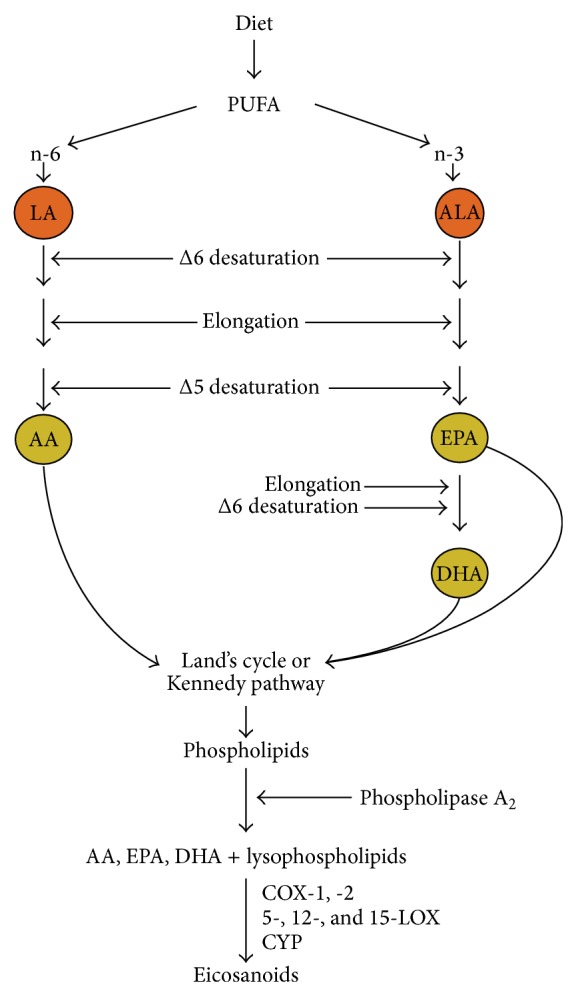
Simplified diagram of eicosanoid synthesis. The *α*-linolenic and linoleic acids are substrates for the synthesis of AA, EPA, and DHA which are added at the sn-2 position to lysophospholipids (via the Lands cycle) or to glycerophospholipids (via the Kennedy pathway). The AAs, EPAs, and DHAs that were released from glycerophospholipids by the PLA(2) are substrates for COX, LOX, cytochrome (CYP), and other enzymes involved in the eicosanoid synthesis.

Because the human body lacks the set of enzymes needed to synthesize the polyunsaturated fatty acids (PUFAs) and *α*-linolenic (ALA) and linoleic acids (LA), diet is their only source. The rate of (PUFAs) conversion into AA, DHA, and EPA is slow due to the low activity of the Δ6 desaturase. That is why only 0,2–2% of the dietary ALA is converted into EPA and DHA, while the rest undergoes *β*-oxidation [1]. The simplest way to bypass this step towards eicosanoid synthesis is to increase the dietary intake of DHA and EPA. Cells also use PUFAs to de novo synthesize glycerophospholipids via the Kennedy pathway or via lysophospholipids remodeling in the Lands cycle [1, 2]. Glycerophospholipids are part of the cell membranes and influence their properties and functions. The next step in eicosanoid synthesis is catalyzed by the phospholipase A_2_ (PLA_2_) with calcium ions and ATP as cofactors and involves the hydrolysis at the sn-2 position of glycerophospholipids. The products of this reaction are free fatty acids and lysophospholipids. The AA, DHA, and EPA released by this reaction are converted into biologically active eicosanoids by the 5-, 12-, and 15-lipoxygenase (LOX), cyclooxygenase (COX) -1 and -2, and other enzymes (Figure 1).

Each step of the described reactions in Figure 1 is susceptible to the activity of drugs and other factors that influence the profile and quantity of the synthesized eicosanoids. The vast body of knowledge about the drug interactions with metabolic pathways is beyond the scope of daily medical practice; however it should be taken into account when planning clinical trials. The daily clinical experience with common medication such as nonsteroidal anti-inflammatory drugs (NSAIDs) or the corticosteroids and 5-LOX inhibitors demonstrates the scope of changes in medical practice caused by drugs. For that reason, the precise determination of patient exclusion criteria is just as important in clinical trial design as the criteria of inclusion. It should be noted that, when analyzing the influence of DHA and EPA supplementation on the inflammatory markers, we will obtain very different results among patients who are prescribed medication for asthma, coronary artery disease, and diabetes and those who are taking no medications.

## 2. Absorption of LCPUFAs in the Gastrointestinal Tract

Triglycerides (TGs) are emulsified in the stomach and duodenum by the bile and pancreatic juices and then hydrolyzed by the pancreatic lipase into free fatty acids (FFAs) and monoacylglycerols. The FFAs absorption ratio decreases proportionally to the increasing FFA carbon chain length and hydrophobicity, whereas the ratio increases proportionally to the increasing desaturation [3]. A small part of short-chain fatty acids (SCFAs) diffuse via the intestinal mucosal membrane via the flip-flop mechanism [4]. The rest of SCFAs and all of long-chain FAs (LCFAs) are bound to the lipid raft proteins found in the cell membrane domains and transmitted through the intestinal wall [5]. The FAs absorbed by the enterocytes bind to caveolin-1 and accumulate in the cytoplasmic lipid droplets.

Before exiting the enterocytes, the PUFAs are bound into TGs at the sn-2 position [6]. Chylomicrons resulting from the bonding of TG with cholesterol, phospholipids, and apolipoproteins enter the portal circulation. Several proteins are known to transport FA across cell membranes and their distribution is specific to the tissue type and function. For example, the fatty acid translocase (FAT)/CD 36 has the highest expression in muscle tissue, whereas the enterocytes of the small intestine have the highest activity of fatty acid transport protein 4 (FATP4) which specifically binds PUFAs [7, 8].

A reduction of PUFA absorption from the intestinal lumen is observed in diseases involving the damage to the intestinal mucosa or the reduction of pancreatic lipase and bile acid secretion, such as inflammatory bowel diseases, pancreatitis, liver diseases, intestinal fistulas, and extensive resections of the small intestine. At this moment it is difficult to assess the degree to which the above-mentioned diseases influence the PUFA absorption and eicosanoid synthesis.

## 3. The Influence of PUFA Supply Route on the Rate of LCPUFA and Eicosanoid Synthesis

The main PUFAs substrates for this process are the dietary *α*-linolenic and linoleic acids. Their absorption rate is largely dependent on supply route and physical structure. Thus, the PUFA n-3/n-6 concentration ratio in cell membranes significantly increases after just 48 hours after intravenous administration, whereas after oral administration the concentration increases more slowly due to extra time needed for its absorption in the gastrointestinal tract. The profile of synthesized eicosanoids in tissues generally results from the Δ6 desaturase's greater affinity for *α*-linolenic acid than for linoleic acid [9, 10]. Currently, the typical Western diet has n-6/n-3 ratio of 15 : 1, instead of the recommended 3 : 1 [11]. Raatz et al. demonstrated that the n-3 PUFAs absorbtion in the form of lipid emulsion intravenously is faster than oral fish oil capsules [12]. FA emulsification and absorption are impaired in pancreatic insufficiency, inflammatory bowel diseases, postgastrectomy states, intestinal fistulas, and extensive bowel resections [13, 14].

## 4. Transcellular Biosynthesis of Eicosanoids

Although eicosanoids can theoretically be synthesized in one cell, it is observed that* in vivo* this process occurs sequentially in different cell types, for example, blood, endothelial, and connective tissue cells [15, 16]. The intermediate from LCPUFA (e.g., PGH_2_ or leukotriene A_4_) from a single donor cell is transported to an acceptor cell which synthesizes the final product [17, 18]. To this date it has not been explained why this process occurs—after all each cell contains a full set of enzymes needed to complete the synthesis. It is even more surprising because the lipophilicity of these products makes their transport across membranes more difficult [19, 20].

Besides the change in cell number, another factor affecting the eicosanoid synthesis is the maturity of cells. In states of intense catabolism, severe infection, and sepsis and in neoplastic disease the bone marrow releases myeloid-derived suppressor cells (MDSCs). The MDSCs can be subdivided into two major groups: immature granulocytes MDSC (G-MDSC) and monocytes MDSC (M-MDSC) released from the bone marrow into the peripheral bloodstream. In order to suppress immune function, the G-MDSCs primarily use reactive oxygen species (ROS), whereas the M-MDSCs use nitric oxide synthase (iNOS) and arginase [21–24]. The intensity of MDSC-induced immunosuppression dynamically changes with the patient's state. The activity of MDSC leads to arginine starvation, lowering of the proliferation rate, and loss of the T-cell-receptor- (TCR-) associated CD3 *ζ* chain [25, 26]. Besides the arginine starvation in tissues, immunosuppression may be triggered by glutamine deficiency. A deficiency of these amino acids can be expected in undernourished or septic patients as well as during intense catabolic states, for example, after large surgical procedures or posttrauma [27]. The assessment of PUFA supplementation's influence on the inflammatory reaction may be complicated by the immaturity of the immune system cells or amino acid deficiency [28, 29].

## 5. Factors Inhibiting the Δ6 Desaturase Activity

Δ6 desaturase catalyzes the conversion of LA and ALA into AA, EPA, and DHA. Several studies on animal models made in the 90s of the last century, mainly on rats, demonstrated that this conversion is greater in females [30, 31] and decreases due to age [32–34], metabolic syndrome, diabetes [35, 36], and deficiencies of folic acid, zinc [37, 38], and vitamins B_6_, B_12_ [39, 40], and A [41]. In addition, Δ6 desaturase activity is decreased by alcohol [42]. The above-mentioned factors may significantly alter the results of clinical trials on the role of eicosanoids in the inflammatory reaction.

## 6. Factors Modifying the Activity of Phospholipase A_**2**_ (PLA_**2**_)

A very important step in eicosanoid synthesis is the hydrolysis of the membrane glycerophospholipids at the n-2 position by PLA2 into free PUFAs and lysophospholipids. The efficiency of this reaction determines the rate of eicosanoid synthesis. Many factors such as thrombin, angiotensin II, and interleukin-2 influence the activity of PLA2 [43–45]. It is decreased in neoplasms associated with the human epidermal growth factor (HER2) overexpression as well as under the influence of angiotensin II receptor inhibitors, used in the treatment of arterial hypertension, and thrombin inhibitors commonly used in the treatment and prevention of the venous thromboembolic disease [46–48]. Glucocorticoids increase the synthesis of lipocortin and annexin, strong inhibitors of PLA2 activity, leading to the inhibition of eicosanoid synthesis [49–52].

The glycerophospholipid deacylation/reacylation cycle known as the Lands cycle is responsible for the continuous change of cell membrane composition and properties [53]. After the PLA_2_-catalyzed deacylation of phospholipids, the lysophospholipid acyltransferases (LPAATs) catalyze the reacylation of lysophospholipids [54, 55]. The efficiency of reacylation of lysophospholipids is influenced primarily by the availability of active PUFA (PUFA-CoA) and the free L-carnitine concentration, which by binding a significant amount of PUFA limits the rate of this step of eicosanoid synthesis [56, 57].

## 7. Factors Influencing COX Activity

NSAIDs inhibit eicosanoid synthesis by acting on COX-1 and -2. Aspirin differs from the other NSAIDs due to its ability to irreversibly acetylate COX-2 and switch this enzyme to instead generate 15R-HETE, a substrate for the 5-LOX. This results in the synthesis of 15-epi-lipoxin A_4_, also known as aspirin-triggered lipoxin (ATL) [58]. Celecoxib and rofecoxib inhibit the activity of acetylated COX-2 and the synthesis of the anti-inflammatory 15(R)-epi-LXA4; therefore combining them with aspirin significantly increases the risk of gastric mucosa damage [59]. These adverse interactions have not been observed after the administration of celecoxib and rofecoxib with the new acetylsalicylic acid derivative NCX 4016 [60]. The acetylated COX-2 converts EPA into 18(R)-hydroxyeicosapentaenoic acid (18R-HEPE) which is then converted by 5-LOX into resolvin (Rv) E_1_ and RvE_2_. RvE_1_ exerts its anti-inflammatory activity after binding with the ChemR23 and leukotriene BLT_1_ receptors [61, 62]. The synthesis of D-series Rv may take place via two pathways. The first of them is dependent on the aspirin-acetylated COX-2 and uses DHA as a substrate for 17(R)-hydroxy-DHA which later is converted by LOX to 17(R)-RvD1 to D4, known as the aspirin-triggered RvD-series (AT-RvDs) [63, 64], whereas the second pathway is independent of aspirin and yields resolvins similar to those from the first pathway.

Although statins use a slightly different mechanism than aspirin to modify COX-2 activity, both drugs produce 15(R) HETE which is converted by the 5-LOX to 15(R)-epi-LXA4 [65, 66]. Clinical studies of the DHA's and EPA's effect on the inflammatory reaction need to consider the fact that aspirin, NSAIDs, and statins all significantly change the eicosanoid profile (Equation (1)) [67].

Aspirin- (ASA-) induced acetylation of cyclooxygenase-2 (COX-2) alters enzyme's specificity as follows:

(1) AA→COX2+ASA15R-HETE→5-LOX15-epi- LXA4 +15-epi- LXB4AA→COX2+ASA+NSAIDs0DHA→COX2+ASAAT-PD1+AT-RvD (1–4)EPA→COX2+ASA18R-HEPE→5-LOXresolvin  E1RvE1and  resolvin  E2RvE2

Aspirin- (ASA-) induced acetylation of cyclooxygenase-2 (COX-2) alters the enzyme specificity changing the outcome of the catalytic reaction with fatty acids leading to the so-called “aspirin-triggered (AT)” products instead. Arachidonic acid (AA) gets converted into 15R hydroxyeicosatetraenoic acid (15R-HETE), which in the presence of 5-LOX transforms further into 15-epi-lipoxins (15-epiLX) known as ATLXA4 and ATLXB4. Docosahexaenoic acid (DHA) produces aspirin-triggered resolvins and protectin D, ATRvD1 to ATRvD4 and ATPD, while reaction with eicosapentaenoic acid (EPA) results in resolvins E, ATRvE1 and ATRvE2. However certain NSAIDs can block these reactions.

## 8. Factors Influencing the 5-, 12-, and 15-LOX Activity

Leukotrienes (LTs) generated by 5-LOX play a key role in the pathogenesis of asthma, allergic rhinitis, and other diseases [68]. Inhibitors of this enzyme and the cysteinyl leukotriene receptor antagonists (e.g., zafirlukast, montelukast, pabilukast, and pranlukast) are successfully used to prevent the exacerbations of those diseases. The 5-LOX inhibitors are not useful in the treatment of asthmatic attacks because the inhibition of LT synthesis also inhibits the transcellular synthesis of the proresolving substances such as resolvins, protectins, and maresins [69]. Pergola et al. demonstrated that the low levels of testosterone in women are the reason for their nearly double higher level of 5-LOX products than in men [70, 71]. This observation explains why the treatment and asthma symptoms control are more difficult in women than in men.

## 9. Conclusions

Laboratory research on PUFAs metabolism is devoid of the confounding factors that exist in clinical practice: age, sex, social conditions, coexisting diseases, and current medication. All of the above factors change the course of numerous chemical reactions and metabolic pathways. We suggest that one of the reasons for the inconsistencies between the results of laboratory and clinical research might be imprecise patient inclusion and exclusion criteria. In this paper we presented information that is infrequently described in medical literature: some of the factors modulating the eicosanoid synthesis and the resultant inflammatory reaction. We hope that this information will help reduce the flaws at the study design stage of clinical trials regarding the PUFA supplementation.
